# Liver fibrosis promotes immunity escape but limits the size of liver tumor in a rat orthotopic transplantation model

**DOI:** 10.1038/s41598-021-02155-9

**Published:** 2021-11-24

**Authors:** Tongqiang Li, Jiacheng Liu, Yingliang Wang, Chen Zhou, Qin Shi, Songjiang Huang, Chongtu Yang, Yang Chen, Yaowei Bai, Bin Xiong

**Affiliations:** 1 Department of Radiology, Union Hospital, Tongji Medical College, Huazhong University of Science and Technology, Jiefang Avenue #1277, Wuhan, 430022 China; 2 Hubei Province Key Laboratory of Molecular Imaging, Wuhan, 430022 China

**Keywords:** Medical research, Oncology, Risk factors

## Abstract

Liver fibrosis plays a crucial role in promoting tumor immune escape and tumor aggressiveness for liver cancer. However, an interesting phenomenon is that the tumor size of liver cancer patients with liver fibrosis is smaller than that of patients without liver fibrosis. In this study, 16 SD rats were used to establish orthotopic liver tumor transplantation models with Walker-256 cell lines, respectively on the fibrotic liver (n = 8, LF group) and normal liver (n = 8, control group). MRI (magnetic resonance imaging) was used to monitor the size of the tumors. All rats were executed at the third week after modeling, and the immunohistochemical staining was used to reflect the changes in the tumor microenvironment. The results showed that, compared to the control group, the PD-L1 (programmed cell death protein receptor-L1) expression was higher, and the neutrophil infiltration increased while the effector (CD8+) T cell infiltration decreased in the LF group. Additionally, the expression of MMP-9 (matrix metalloproteinase-9) of tumor tissue in the LF group increased. Three weeks after modeling, the size of tumors in the LF group was significantly smaller than that in the control group (382.47 ± 195.06 mm^3^ vs. 1736.21 ± 657.25 mm^3^, P < 0.001). Taken together, we concluded that liver fibrosis facilitated tumor immunity escape but limited the expansion of tumor size.

## Introduction

A distinct feature of hepatocellular carcinoma (HCC) is that it is closely related to liver fibrosis, and 80–90% of HCC occurred in the fibrotic or cirrhotic liver^1,2^. The complex pathogenesis of liver fibrosis includes activation and recruitment of immunecells, activation of hepatic stellate cells (HSCs) and hepatic myofibroblasts (MFs), and the synthesis of fibrotic extracellular matrix (ECM)^3–5^.

Several studies have revealed that the tumor immunity escape and angiogenesis promoted by liver fibrosis can affect the occurrence, development and recurrence of HCC^6–8^. However, strangely, compared with the HCC patients without cirrhosis, patients with cirrhosis have smaller tumor size^6,9,10^. As far as we know, one of the main consequences of liver fibrosis is the increased stiffness, and there is a linear relationship between the severity of liver fibrosis and liver stiffness^11,12^. And the physical tension produced by collagen deposition which came from MFs may restrict the growth space of the tumor^13^. Despite these theories, there is no literature to describe the relationship between tumor size and liver fibrosis.

In this study, we established a rat orthotopic transplantation model with a liver fibrosis background, verified whether liver fibrosis causes tumor immunosuppression, and further explored the effect of liver fibrosis on tumor growth.

## Materials and methods

### Animals

16 male SD rats (280–320 g) were purchased from the Experimental Animal Center of Tongji Medical College at University of Science and Technology. All experimental methods were approved by the Ethics Committee of Tongji Medical College at Huazhong University of Science and Technology. The experimental methods were carried out in accordance with the appropriate approvals and relevant guidelines. The rats were maintained in a SPF environment, with free access to food and water. Rats were euthanized with CO_2_, followed by neck dislocation. This study complies with the ARRIVE guidelines.

### The establishment of different liver background

**Figure 1:**
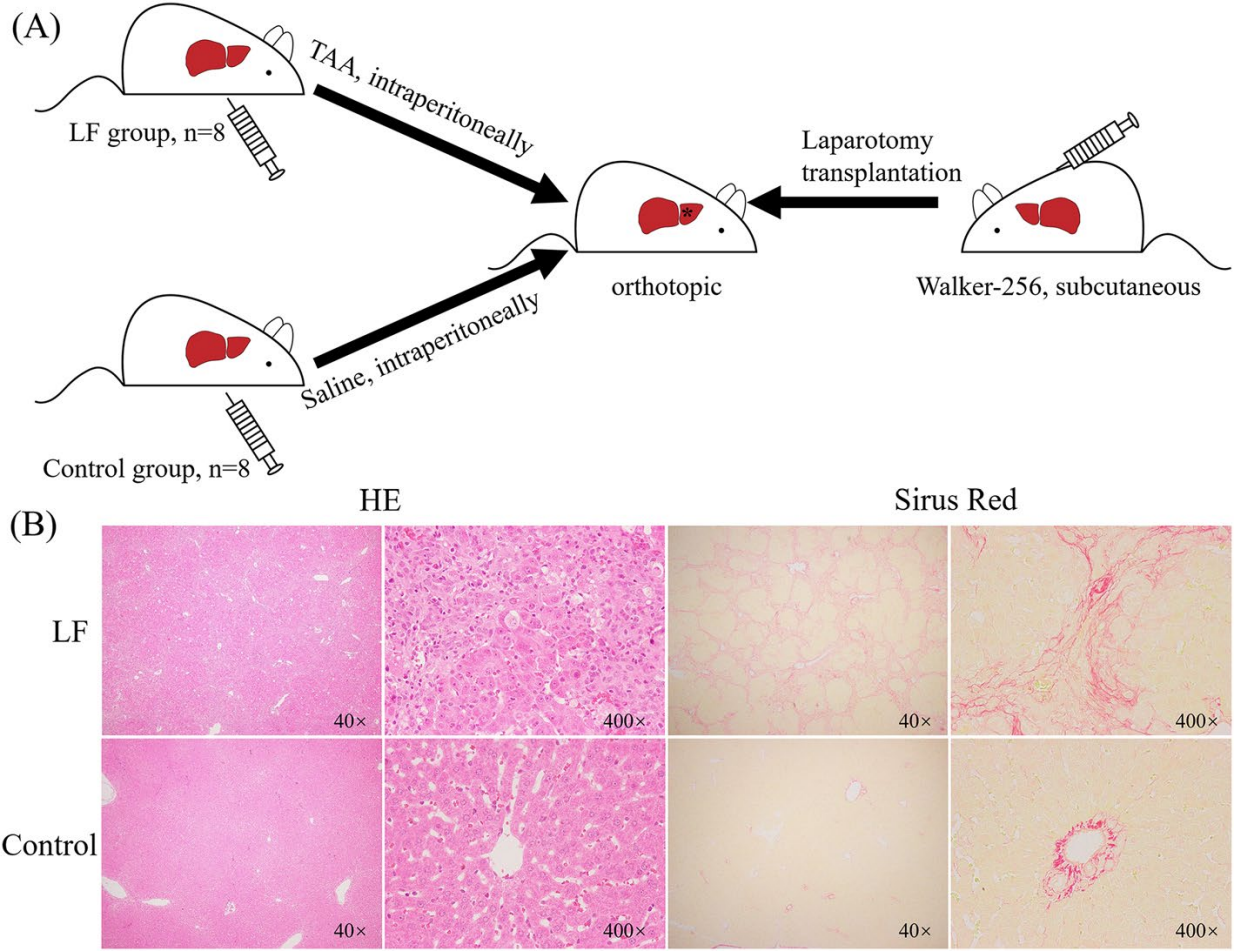
(**A**) The establishment of rat orthotopic transplantation model in LF and control group; (**B**) Comparison of HE and Sirius Red staining (40× and 400×) of the liver in LF and control group.

16 rats were randomly divided into two groups. Liver fibrosis was induced with the method which was described in a previous study^14^. Specifically, 8 rats were treated thrice per week for the LF group with intraperitoneal injections of 250 mg/kg Thioacetamide (TAA) for 6 weeks. Correspondingly, 8 rats were injected with isometric normal saline for the control group (Fig. 1A).

### The establishment of rat liver tumor orthotopic transplantation model

We chose Walker-256 cells (Procell Medical Co. Ltd., Wuhan, China) as the allograft cell line because it can observe the changes in the tumor microenvironment^15,16^, and the number of experimental animals can be reduced due to its high tumor formation rate. To obtain the tumor mass, 1 ml cell suspension containing 1 × 10^6^ cells was subcutaneously injected into the right flank of a rat (tumor-bearing rat). When the subcutaneous tumor reached 1 cm in length, the tumor-bearing rat was sacrificed and the tumor tissue was removed for orthotopic transplantation in experimental rats (Fig. 1A).

The method of open orthotopic transplantation was described in the previous literatures^17,18^. Briefly, the fresh tumor tissue was cut and separated into cubes at the size of 1 mm^3^ under sterile conditions and then these pieces were stored in saline. Next, under 1% isopentobarbital sodium anesthesia, an incision was made in rats along the abdominal white line. Under aseptic conditions, a tumor piece was embedded into each rat's hepatic left lateral lobe and then blocked with a gelatin sponge to prevent the tumor mass from falling out or liver bleeding. Finally, the wound was sutured after ensuring no bleeding or complications.

### MRI scan acquisition

All rats were monitored by a 3T MR system (PHILIPS, Holland) with an eight‐channel phased‐array coil designed for rats (Medcoil Healthcare, Suzhou, China) in the 1, 2 and 3 weeks after modeling. The T2WI-TSE images were obtained using a field of view (FOV) of 100 × 60 mm, 256 × 196 matrix, 25 slices of 1 mm thickness, repetition time = 2500 ms, and TE = 100 ms. The long diameter (a) and short diameter (b) of the tumor were measured independently by two experienced radiologists who did not know the rat grouping. The tumor volume was calculated as V = a × b^2^/2, and the tumor growth rate was calculated as V_n_ /V_1_ × 100% (V_n_ represented the tumor volume at the n week, n = 2, 3).

### Sample collection

In week 3, all rats were sacrificed immediately after the MRI scan, then the liver and tumor tissue was removed and preserved in 4% formaldehyde for 24 h before paraffin-embedded sections were made.

### Hematoxylin–Eosin staining and Sirius Red staining

A 4-μm paraffin serial sections of the liver per rat were stained with Hematoxylin–Eosin (HE), with Sirius Red for collagen to evaluate the collagen deposition of liver fibrosis.

### Immunohistochemical (IHC) staining

IHC staining was performed on formalin-fixed, paraffin-embedded tumor samples and the method was as described in the previous literature^19^. Briefly, paraffin sections were taken and dewaxed to water. Antigen repair solutions were dripped on the sections and washed with PBS 3 times. The first antibodies were added to the sections and washed with PBS (PH7.4) 3 times at 4 °C overnight. Secondary antibodies were added and rinsed with PBS 3 times again. Immunostaining was performed with DAB. The sections were counterstained with hematoxylin. The antibodies Ki67 (1:200, Abcam), CD31 (1:400, DAKO, USA), α-SMA (1:500, Themo Fisher), PD-L1 (1:600, Abcam), CD8 (1:2000, NOVUS, USA), Ly6G (1:800, Servicebio), MMP-2 (matrix metalloproteinase-2, 1:1500, Servicebio) and MMP-9 (1:800, Servicebio) were used. Visualize staining of tissue under a microscope, acquisitive and analysis image (Nikon DS-U3, Japan). The results of IHC staining were analyzed by Image J software 1.8.0 (Media Cybernetics, Rockville, MD, USA). The Ki67 and CD8 antibody staining results were evaluated by the percentage of positive cells and the positive cells density respectively, and the percentage of positive staining areas evaluated the α-SMA, CD31, PD-L1, Ly6G, MMP-2 and MMP-9 antibody staining results. Five random visual fields were counted for each sample and the average was determined.

### Statistical analysis

Statistical analysis was done with SPSS 24.0 (SPSS Inc., Chicago, USA) and GraphPad Prism 8.0 (GraphPad Software, La Jolla, USA) software. The data were described as mean value ± standard deviation or frequency (percentages). Calibrated Chi-square test and unpaired t-tests were applied, as well as the Fisher exact test. Spearman’s correlation test was used for correlation analysis. P value < 0.05 was considered significant.

## Results

### All rats were successfully modeled

As shown in Fig. 1B, the HE and Sirius red staining of the liver in the LF group indicated that the morphology of the liver lobules was disordered, large amounts of collagen deposition formed, and there was fibrosis between the vascular areas. Additionally, as expected, MMPs of liver tissue were abundantly expressed in the LF group (Fig. 4B,C). Based on these, we deemed that the rats in the LF group suffered liver fibrosis from TAA injection was stopped until they were sacrificed, while the liver anatomy of the control group showed no apparent abnormalities. Furthermore, we observed that although given the same dose of TAA simultaneously, the degree of fibrosis between rats in the LF group was slightly different due to the individual divergence.

### Liver fibrosis promoted immunity escape and tumor angiogenesis

**Figure 2:**
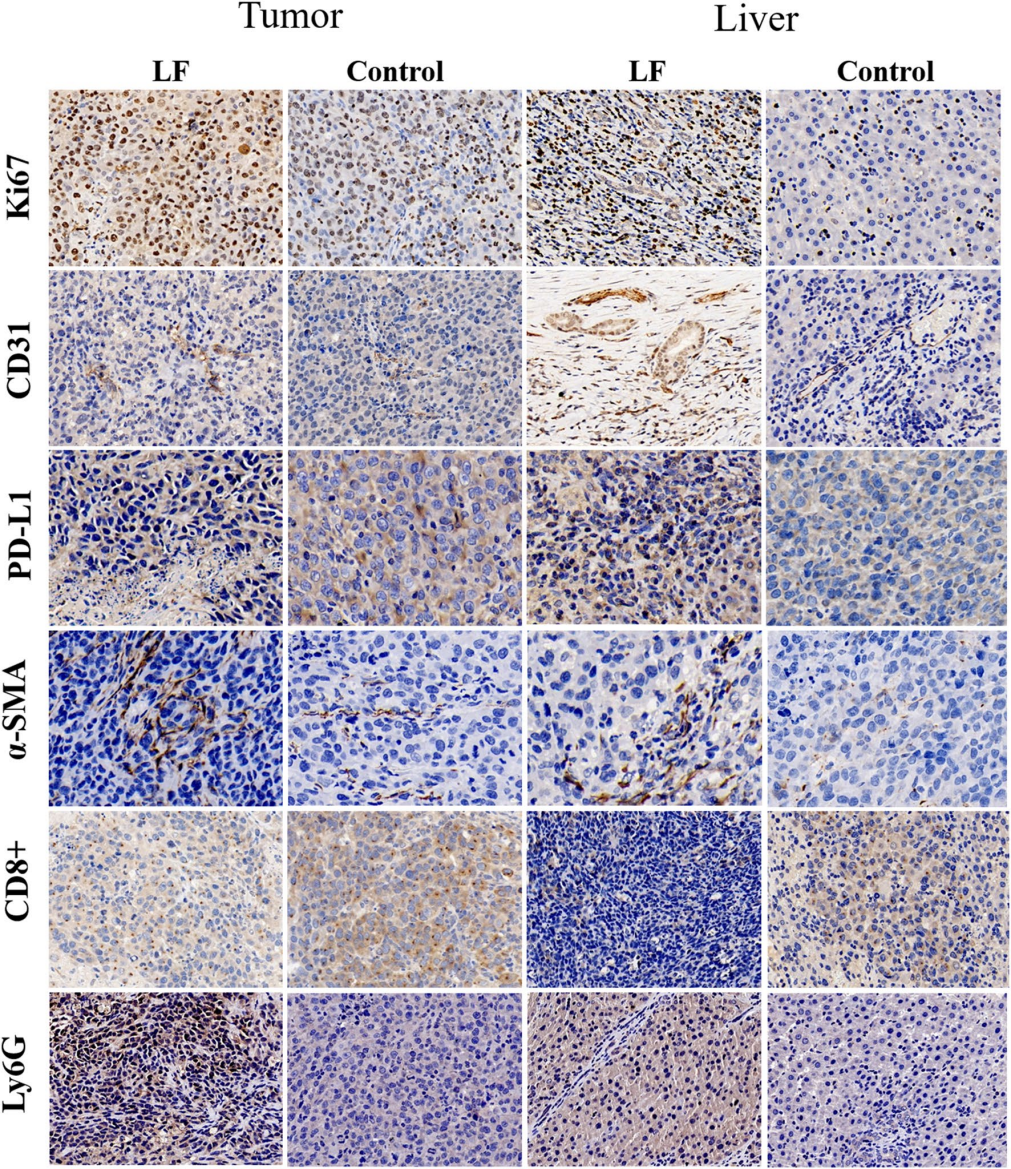
Representative pictures of IHC staining of tumor and liver in LF group and control group (400×).

**Figure 3:**
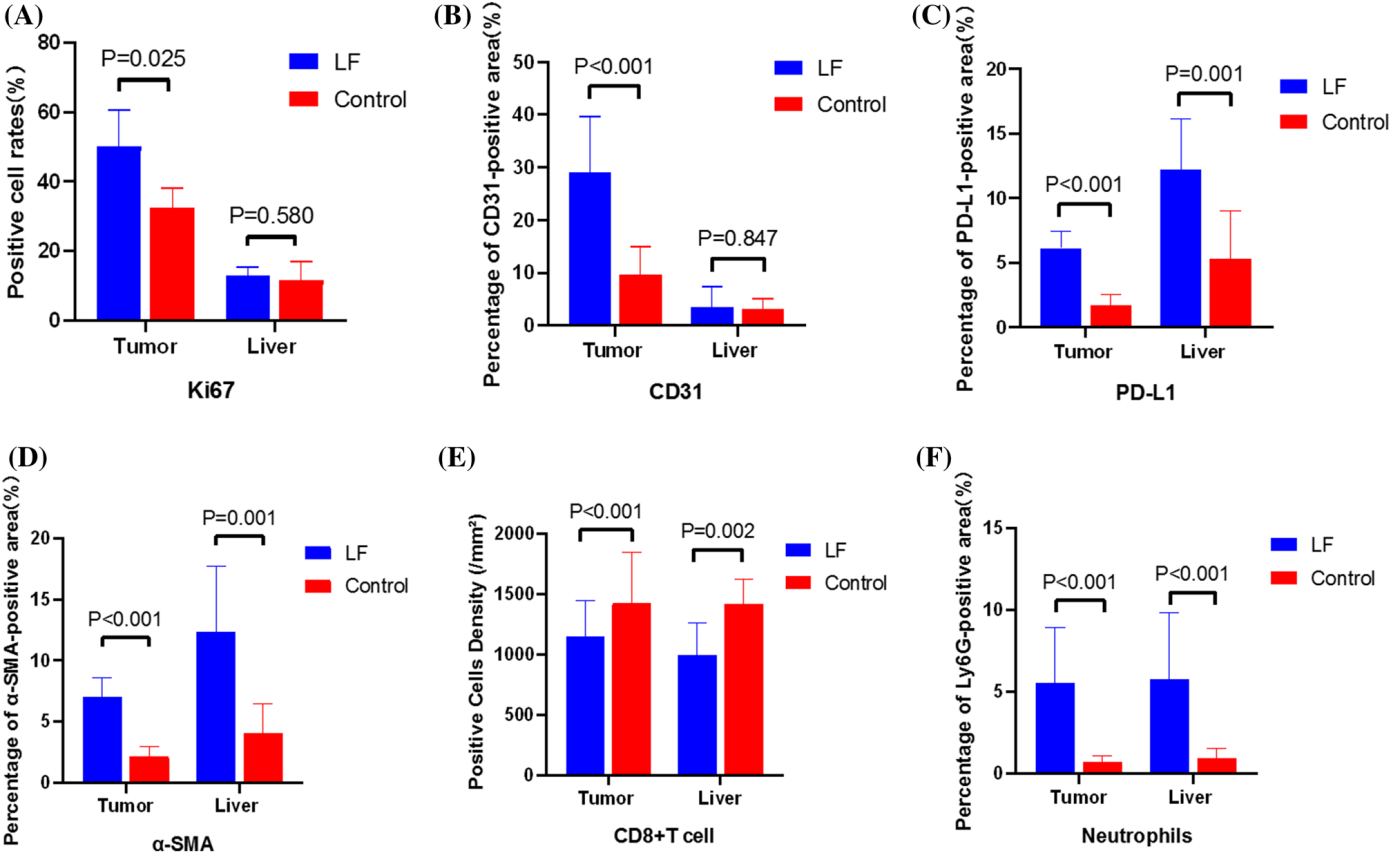
Quantitative analysis of immunohistochemistry results of Ki67, CD31, PD-L1, α-SMA, CD8 + T cell and Ly6G.

We simultaneously observed the IHC results of the tumor and adjacent liver tissue. The presentative pictures of the IHC staining of the tumor and liver were shown in Fig. 2. In tumor tissues, compared to the control group, the PD-L1 expression was significantly higher (P < 0.001, Fig. 3C), the neutrophil infiltration increased (P < 0.001, Fig. 3F) while the CD8 + T cell density decreased (P < 0.001, Fig. 3E) in the LF group. The MVD marked by CD31 in the LF group was higher than that in the control group (P < 0.001, Fig. 3B) and the cell proliferation represented by Ki67 expression was also significantly different (P = 0.025, Fig. 3A). These pieces of evidence showed that tumors in the LF group experienced immunosuppression, and tumor angiogenesis and tumor cell proliferation increased. Although there is still controversy, cancer-associated myofibroblasts (CAFs) are widely considered to be derived from HSCs, and α-SMA is one of the markers of CAFs^20,21^. It made sense that a large number of activated HSCs in adjacent liver tissues infiltrated into the tumor and became a part of the tumor immune microenvironment (Fig. 3D)^22^. Although the liver tissue suffered immunosuppression which was similar to tumor tissue, which to a certain extent corroborated the changes in precancerous microenvironment (PME), there is no difference between the LF group and control group in the expression of Ki67 and CD31 (Fig. 3A,B).

**Figure 4:**
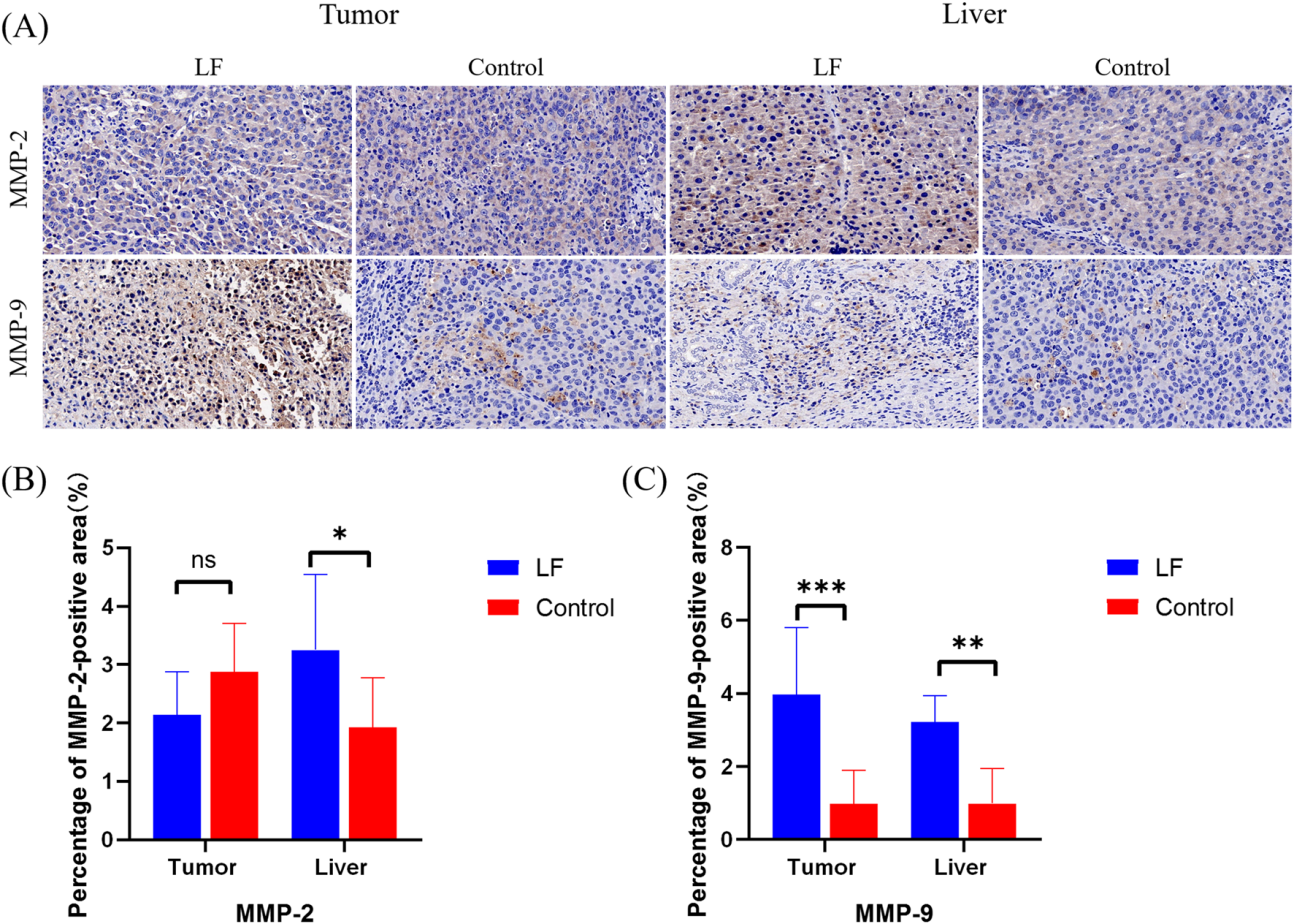
(**A**) Representative pictures of IHC staining of MMP-2 and MMP-9 in LF group and control group (400×); (**B**), (**C**) Quantitative analysis of immunohistochemistry results of MMP-2 and MMP-9. *P < 0.05; **P < 0.01; ***P < 0.001.

Similar to the previous studies, after TAA injection, the expression of MMP-2 and MMP-9 in the liver of the LF group increased (Fig. 4A–C)^23^. In tumor tissue, we found that the expression of MMP-2 was not statistically significant between the two groups (Fig. 4B), while the expression of MMP-9 in the LF group was higher than that in the control group (Fig. 4C).

**Figure 5:**
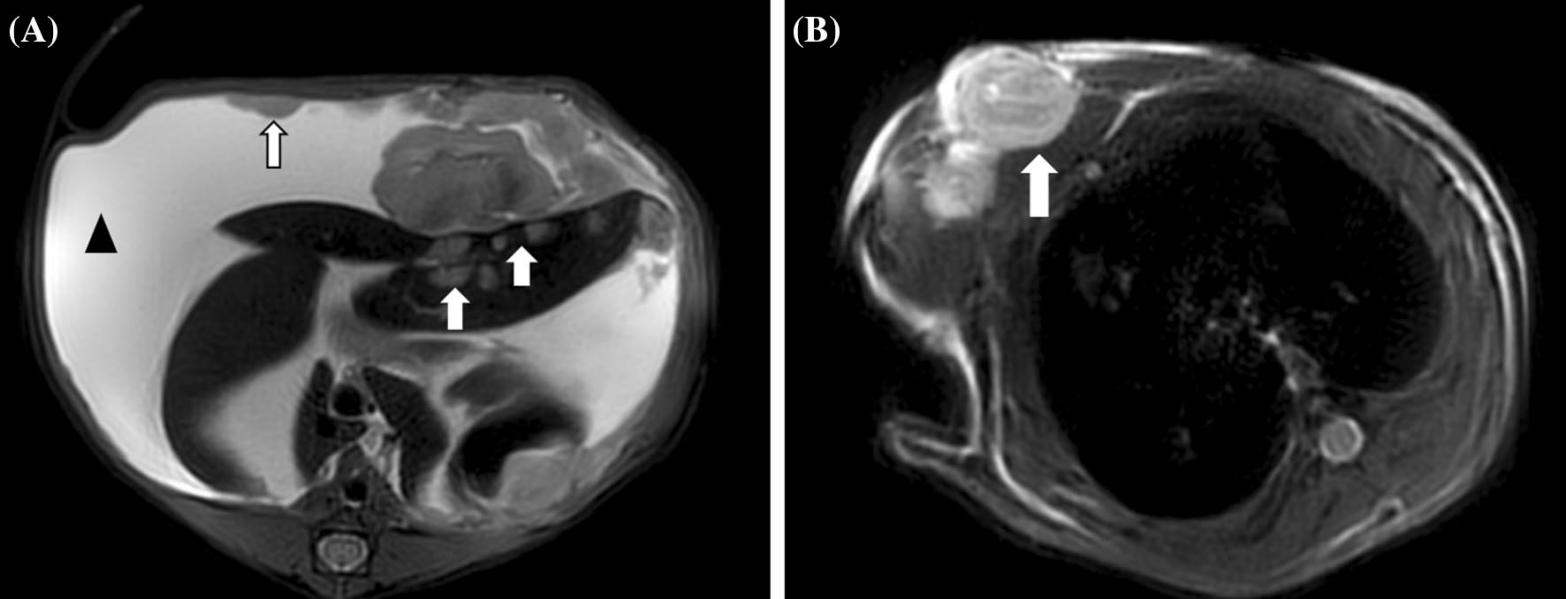
(**A**) In the third week, T2WI image of a rat in LF group showing intrahepatic metastasis (white arrow), ascites (black arrow) and epigastric metastasis (white arrow); (**B**) T2WI image showing chest-wall metastasis (white arrow).

In addition, we also observed the metastasis of tumors on MRI. The intrahepatic metastasis was defined as metastasis confined to the liver (Fig. 5A), and the extrahepatic metastasis including epigastric and chest-wall metastasis (Fig. 5B). Although there was no statistical difference, the tumors in the LF group were generally more likely to metastasize than those in the control group (Table 1).

**Table 1:** Tumor metastasis in LF group and control group.

Time	Metastasis	LF^a^	Control^a^	P value
1w	Intrahepatic metastasis	1/8	0/8	/
	Extrahepatic metastasis	0/8	0/8	/
2w	Intrahepatic metastasis	3/8	2/8	/
	Extrahepatic metastasis	2/8	1/8	/
3w	Intrahepatic metastasis	4/8	2/8	0.608
	Extrahepatic metastasis	5/8	2/8	0.315

^a^n/8 means that n out of 8 rats have metastasized at the given moment.

### Tumor size in LF group was smaller

**Figure 6:**
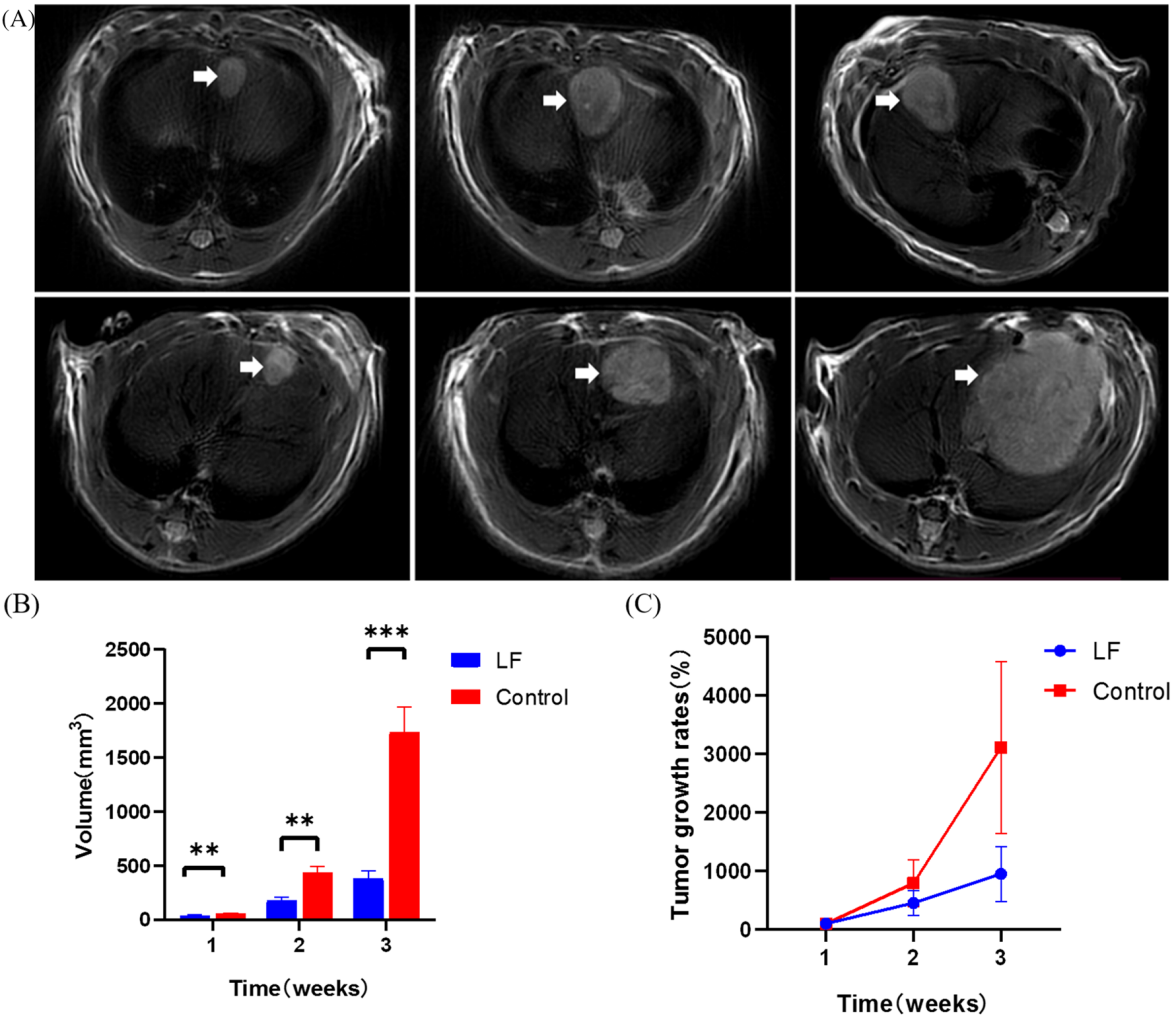
(**A**) MR dynamic detection of LF group and control group, tumor tissue showed high signal in T2WI sequence (white arrow); (**B**) Tumor volume changes of LF group and control group; (**C**) The tumor growth rates of LF group and control group. **P < 0.01; ***P < 0.001.

The tumors were presented clearly on MRI (Fig. 6A). The tumor volume of the LF group and control group in the 1–3 weeks was: 41.43 ± 8.11 mm^3^ vs. 58.22 ± 9.26 mm^3^ (P = 0.002), 181.66 ± 79.41 mm^3^ vs. 438.06 ± 163.21 mm^3^ (P = 0.001), 382.47 ± 195.06 mm^3^ vs. 1736.21 ± 657.25 mm^3^ (P < 0.001) (Fig. 6B). The tumor growth rates of the LF group and control group at week 2 and 3 were 457.01 ± 207.11% vs. 795.43 ± 396.70% (P = 0.051) and 951.43 ± 470.42% vs. 3118.01 ± 1468.32% (P = 0.001) (Fig. 6C).

We measured the degree of collagen deposition of all rats’ livers, which was quantified by the percentage of collagen area in the sirius red staining (Fig. 1B). Then the spearman’s correlation test was used to analyze the correlation between tumor size and liver collagen deposition (Table 2). These data showed that tumor volume was strongly correlated with the percentage of collagen area (r = − 0.823, P < 0.001, n = 16). It was worth mentioning that, more CD8 + T cells and fewer neutrophils in the tumor, the more giant the tumor (Table 2).

**Table 2:** The spearman’s correlation analysis between tumor size and tumor immunosuppressive or liver collagen deposition (n = 16).

Tissue	Independent variable	Spearman r*	P value
Tumor	Ki67	**− 0.735**	**0.001**
	CD31	**− 0.591**	**0.016**
	PD-L1	**− 0.650**	**0.006**
	α-SMA	**− 0.779**	**< 0.001**
	CD8 + T cell	**0.670**	**0.005**
	Neutrophils	**− 0.662**	**0.005**
Liver	Ki67	**− **0.294	0.269
	CD31	0.285	0.284
	PD-L1	**− **0.397	0.128
	α-SMA	**− **0.512	0.043
	CD8 + T cell	0.525	0.037
	Neutrophils	**− **0.679	0.004
	collagen	**− 0.823**	**< 0.001**

Significant values are in bold.

r: 0–0.3, uncorrelated; 0.3–0.6, weakly correlated; 0.6–0.8, moderately correlated; > 0.8, strongly correlated.

## Discussion

The impact of liver fibrosis on the growth of HCC has attracted significant attention in recent years because of the prevalence of liver cancer patients with cirrhosis^24–26^. Much research has focused on the impact of liver fibrosis on the tumor microenvironment^24,27–29^, but few studies focused on the effect of liver fibrosis on tumor size. In fact, the tumor size is a critical yardstick for determining suitable treatment and assessing treatment responses for clinicians^9,30^. And an interesting phenomenon is that the size of the tumor with liver fibrosis does not match the immune escape characteristics. In this current study, we observed the effect of liver fibrosis on the growth of liver tumors in a rat orthotopic transplantation model, and we found that although the size of the tumors in the LF group was smaller, they had a stronger immunosuppressive state and aggressiveness.

Many aspects of liver fibrosis can be involved in promoting immune escape for tumors. The activation of HSCs is considered the core event in developing liver fibrosis and final cirrhosis^8,31^. In addition to directly affect the development of HCC by secreting key cytokines and chemokines, such as HGF, TGF-β, PDGF, interleukin-6 and Wnt ligands^24,32,33^, the activated HSCs also secrete vascular endothelial growth factor (VEGF), CXC chemokine to promote vascular and actively participates in the occurrence and development of tumor blood vessels remodeling^8,27,34^. In addition, activated HSCs also exhibit immunomodulatory activity by expressing proteins such as PD-L1 and B7-H4, and promoting the expansion of immunosuppressive cells such as regulatory T cells (Tregs) and myeloid-derived suppressor cells (MDSC)^26,35–37^.

The accumulation of collagens, predominantly type I collagen, resulting in a two to five fold increase of total collagen content in the cirrhotic liver^1,38^. Several of the ECM components such as collagens, laminins, fibronectin, glycosaminoglycans and proteoglycans interact directly and indirectly with HCC cells and the stroma cell types, thereby changing the tumor microenvironment^39^. These ECM proteins also store growth factors such as HGF, PDGF, TGF-β, CTGF, and VEGF, which influence the immunity escape of tumor environment^1^. Changes in the biomechanical environment of HCC can transmit signals to HCC cells through mechanoreceptors such as integrin, which activates signal pathways such as YAP/TAZ and promotes the proliferation, invasion and metastasis of HCC^28,39–41^.

Tumor-associated neutrophils (TANs) in hepatocellular carcinoma received attention in recent years. TGF-β, which was secreted by activated HSCs, plays a major role in neutrophil plasticity, driving the acquisition of an N2 phenotype^42^. The N2 TANs have been proved that they can recruit macrophages and Treg cells into HCCs to promote their growth, progression^43^. In the present study, we did observed that the TANs infiltration in the LF group was higher than that in the control group. At the same time, the immunosuppression of liver fibrosis on tumors was also reflected in the increased expression of PD-L1 and decreased CD8 + T cell infiltration in the LF group.

MMPs are calcium-dependent zinc-containing peptidases and are responsible for the degradation and turnover of most components in the ECM during fibrogenesis^44^. MMPs also have other functions besides participating in ECM turnover, including regulating signaling pathways that control cell growth, inflammation, or angiogenesis and may even work in a nonproteolytic manner^5^. MMP-2 and MMP-9 are two key MMPs secreted from HSCs and have been proved highly expression during TAA induced fibrogenesis^23,45,46^. High expression of MMP-2, MMP-9, and both has been associated with tumor progression and poor survival of HCC patients^47^. Overexpression of MMPs in tumor cells will enhance degradation of the basement membrane to facilitate invasion of nearby blood vessels, followed by extravasion to distant tissues to seed new metastatic sites and tumor cells mainly express MMP-9 instead of MMP-2 in HCC^47^. In the tumor tissue of LF group, we observed that the expression of MMP-9 was higher than that of control group in liver tissue while there was no statistical difference in the MMP-2 expression between two groups, which may be related to the characteristics of tumor cell lines.

The harder the liver, the smaller the space for tumor growth, which was reflected in this study's negative linear relationship between collagen deposition and tumor size. Nevertheless, this does not mean that liver fibrosis can reduce the degree of “tumor damage”. In fact, despite the smaller tumor size, the HCC patients with cirrhosis have a worse prognosis and higher-level pathological typing^6,9^. Liver stiffness is positively correlated with the risk of HCC, patients with a liver stiffness value greater than 12.5 to 13 kPa have a 4 to 13 times higher risk of HCC occurrence^48,49^. Schrader et al.^50^ found that when cells were cultured on hard (12 kPa) supports, the proliferation index (assessed by Ki67) of Huh7 and HepG2 cells were respectively 2.7 and 12.2 times higher than those were cultured on soft (1 kPa) supports.

In addition, we also found a relatively moderate correlation between tumor volume and related indicators reflecting immune escape. For patients who accepted immunity therapy, an apparent initial increase in tumor burden may be present, a finding that is likely related to transient immunity cell infiltration^51^. Moreover, CD8 + T cell infiltration was related to tumor size in this study, but more shreds of evidence are needed to prove the relationship between tumor size and immunity cells^51,52^.

Different from the previous focus on the effect of stiffness on the biological behavior of tumor cells^50,53^, the model we constructed in this study can directly observe the effect of liver fibrosis on tumor size. A limitation to the study is that the number of animals in each group was relatively small, which might reduce statistical efficiency. In summary, liver fibrosis facilitated tumor immunity escape but limited the expansion of tumor size, and this phenomenon may be related to the accumulation of collagen in the liver and the decrease of lymphocyte infiltration in the tumor.
